# Unraveling Specific Causes of Neonatal Mortality Using Minimally Invasive Tissue Sampling: An Observational Study

**DOI:** 10.1093/cid/ciz574

**Published:** 2019-10-09

**Authors:** Shabir A Madhi, Jayani Pathirana, Vicky Baillie, Alane Izu, Quique Bassat, Dianna M Blau, Robert F Breiman, Martin Hale, Azwifarwi Mathunjwa, Roosecelis B Martines, Firdose L Nakwa, Susan Nzenze, Jaume Ordi, Pratima L Raghunathan, Jana M Ritter, Fatima Solomon, Sithembiso Velaphi, Jeannette Wadula, Sherif R Zaki, Richard Chawana

**Affiliations:** 1 Medical Research Council, Respiratory and Meningeal Pathogens Research Unit, University of the Witwatersrand, Faculty of Health Science, Johannesburg, South Africa; 2 Department of Science and Technology/National Research Foundation, Vaccine Preventable Diseases, University of the Witwatersrand, Faculty of Health Sciences, Johannesburg, South Africa; 3 ISGlobal, Hospital Clínic, Universitat de Barcelona, Barcelona, Spain; 4 Centro de Investigação em Saúde de Manhiça (CISM), Maputo, Mozambique; 5 Catalan Institution for Research and Advanced Studies (ICREA), Barcelona, Spain; 6 Pediatric Infectious Diseases Unit, Pediatrics Department, Hospital de Sant Joan de Deu, University of Barcelona, Barcelona, Spain; 7 Consorcio de Investigacion Biomedica en Red de Epidemiologia y Salud, Madrid, Spain; 8 Center for Global Health, Centers for Disease Control and Prevention, Atlanta, Georgia, USA; 9 Emory Global Health Institute, Emory University, Atlanta, Georgia, USA; 10 National Health Laboratory Service, Department of Anatomical Pathology, School of Pathology, University of the Witwatersrand, Faculty of Health Sciences, Johannesburg, South Africa; 11 Infectious Diseases Pathology Branch, Division of High-Consequence Pathogens and Pathology, National Center for Emerging and Zoonotic Infectious Diseases, Centers for Disease Control and Prevention, Atlanta, Georgia, USA; 12 Department of Paediatrics, Chris Hani Baragwanath Academic Hospital, School of Clinical Medicine, Faculty of Health Sciences, University of the Witwatersrand, Johannesburg, South Africa; 13 National Health Laboratory Service, Department of Microbiology and Infectious Diseases, School of Pathology, Faculty of Health Sciences, University of the Witwatersrand, Johannesburg, South Africa

**Keywords:** MITS, immediate cause of death, underlying cause of death, hospital acquired infection, core biopsy

## Abstract

**Background:**

Postmortem minimally invasive tissue sampling (MITS) is a potential alternative to the gold standard complete diagnostic autopsy for identifying specific causes of childhood deaths. We investigated the utility of MITS, interpreted with available clinical data, for attributing underlying and immediate causes of neonatal deaths.

**Methods:**

This prospective, observational pilot study enrolled neonatal deaths at Chris Hani Baragwanath Academic Hospital in Soweto, South Africa. The MITS included needle core-biopsy sampling for histopathology of brain, lung, and liver tissue. Microbiological culture and/or molecular tests were performed on lung, liver, blood, cerebrospinal fluid, and stool samples. The “underlying” and “immediate” causes of death (CoD) were determined for each case by an international panel of 12–15 medical specialists.

**Results:**

We enrolled 153 neonatal deaths, 106 aged 3–28 days. Leading underlying CoD included “complications of prematurity” (52.9%), “complications of intrapartum events” (15.0%), “congenital malformations” (13.1%), and “infection related” (9.8%). Overall, infections were the immediate or underlying CoD in 57.5% (n = 88) of all neonatal deaths, including the immediate CoD in 70.4% (58/81) of neonates with “complications of prematurity” as the underlying cause. Overall, 74.4% of 90 infection-related deaths were hospital acquired, mainly due to multidrug-resistant *Acinetobacter baumannii* (52.2%), *Klebsiella pneumoniae* (22.4%), and *Staphylococcus aureus* (20.9%). *Streptococcus agalactiae* was the most common pathogen (5/15 [33.3%]) among deaths with “infections” as the underlying cause.

**Conclusions:**

MITS has potential to address the knowledge gap on specific causes of neonatal mortality. In our setting, this included the hitherto underrecognized dominant role of hospital-acquired multidrug-resistant bacterial infections as the leading immediate cause of neonatal deaths.

## Teaser

Minimally invasive tissue sampling and clinical history were utilized to attribute cause of death in South African neonates. Leading underlying causes of death were prematurity, complications of intrapartum events, and congenital malformations. The leading immediate cause of death was infection.

In 2017, 47% of an estimated 5.4 million deaths in children aged <5 years occurred within 1 month of birth, with more than three-quarters (77%) occurring in sub-Saharan Africa and South Asia [1]. Currently, causes of under-5 childhood deaths in low- and middle-income countries (LMICs) are mainly inferred from vital registration and limited verbal autopsy data. In 2015, only 3% of under-5 childhood cause-specific mortality fractions (CSMFs) were based on adequate vital registration data, primarily from high-income countries [2]. Inadequacy of vital registration data, coupled with verbal autopsies being available for as few as 1 of every 850 deaths in most LMICs [3], necessitates modeling simulations to impute childhood CSMFs. Furthermore, although verbal autopsies have high concordance in attributing cause of death (CoD) diagnosis compared to physician diagnosis in high-quality hospitals [4], this is only achievable at a broad syndromic level. Also, CSMF is analyzed for the underlying medical condition that led to death, which could undermine recognition of more immediate medical events resulting in death that might be preventable or treatable.

Limitations in identifying the contributory role of infectious causes of deaths is accentuated by scarcity and challenges in antemortem and postmortem investigation of children dying in LMICs [5]. Complete diagnostic autopsy (CDA) is the gold standard for CoD attribution; however, limited pathology capacity, resource constraints, and cultural and religious belief barriers are impediments to undertaking CDA in LMICs [5]. Nevertheless, more refined methods than verbal autopsies are required to elucidate the CoD, which could identify preventable causes and guide empiric treatment [2, 6]. The potential of minimally invasive tissue sampling (MITS) is one such option [7, 8] and is theoretically acceptable (73%) even in LMIC settings [8]. A recent pilot validation study from Mozambique reported moderate concordance between CDA and MITS in 41 neonatal deaths (κ = 0.40 [95% confidence interval, .18–.63), albeit without considering clinical information when attributing the CoD using the MITS data. The concordance between CDA and MITS was higher for deaths attributed to “infections” (85%) or “preterm complications” (60%), while expectedly lower for congenital abnormalities (40%) [9].

The Bill & Melinda Gates Foundation (BMGF) is funding the multicountry Child Health and Mortality Prevention Surveillance (CHAMPS) network, focused on using MITS to ascertain and track the CoD in children in high-mortality areas [6]. As a prelude to the CHAMPS program, we piloted the utility of MITS, interpreted together with available antemortem clinical and laboratory information, to ascertain the causes of stillbirth, neonatal, and childhood deaths in an LMIC setting.

In this manuscript, we report on the utility of MITS for attributing the underlying and/or immediate CoD among neonates in Soweto, South Africa.

## METHODS

Detailed characteristics of the study population, study site, the MITS procedure, laboratory assays, and CoD attribution are provided in the Supplementary Materials, and briefly described here.

### Study Site and Population, Study Design, and Procedures

This prospective, observational pilot study was undertaken at Chris Hani Baragwanath Academic Hospital (CHBAH), the only public hospital in Soweto during the study period (16 July 2015–30 July 2016). Public healthcare is provided free-of-service fee by the State to all pregnant women and children <6 years of age. Although South Africa is a middle-income country, the estimated neonatal mortality rate for Soweto was 22 per 1000 live births (MatFlu Cohort, unpublished data).

Deaths occurring in the neonatal and pediatric medical wards were identified by study staff through reporting by the attending physicians and review of the inpatient ward and mortuary registries throughout the study period, except from 18 December 2015 to 3 January 2016. Also included in the study were children who were dead upon arrival at the hospital. Following identification of the death, study staff approached bereaved parents/guardians to provide grief counseling and inquire about their interest regarding study participation. Parents were provided an opportunity to consult with other family members, including their elders. Study inclusion criteria included birth weight >750 g, residence in Soweto, feasibility of undertaking the MITS within 36 hours after death, and parental consent for participation.

### Minimally Invasive Tissue Sampling

The MITS procedures were undertaken by trained study staff. After the body surface was washed with water and decontaminated using 70% alcohol, multiple brain, lung, and liver tissue samples were collected using core biopsy needles. The tissue samples were sent for histopathological examination (all), culture (lung and liver), and molecular tests (lung). Furthermore, blood and cerebrospinal fluid (CSF) samples were collected for microbiological culture and molecular testing, and rectal swabs for molecular testing. Human immunodeficiency virus (HIV) polymerase chain reaction (PCR) testing (Roche COBAS® TaqMan HIV-1 Qualitative Test Version 2, Roche Molecular Systems, Branchburg, New Jersey) was performed on whole blood samples at the National Health Laboratory Service (NHLS) (Supplementary Table 1).

Blood culture using the BacT/Alert microbial system (bioMérieux, Marcy l’Etoile, France), lung and CSF microbial culture, and antibiotic susceptibility testing were undertaken at NHLS.

All molecular tests were undertaken at the Respiratory and Meningeal Pathogens Research Unit laboratory using commercially available multiplex Fast-Track Diagnostics (FTD, Sliema, Malta) PCR assays, as detailed in the Supplementary Materials.

### Histopathological Diagnosis

Two sets of organ tissue samples were collected from each site; 1 set was processed locally at the NHLS and the other at the Centers for Disease Control and Prevention (CDC) in Atlanta, Georgia. Hematoxylin and eosin (H&E) stains were done and selected specific stains including Ziehl-Neelsen for mycobacteria, Grocott methenamine silver and periodic acid-Schiff for fungi, and Gram stain for bacteria as indicated by the histological findings. Immunohistochemistry was also performed with the choice of antibody guided by the H&E findings, molecular results, and microbiological findings [10].

### Determination of CoD

The CoD was determined by an international panel constituting pathologists, pediatricians, epidemiologists, microbiologists, an obstetrician, infectious disease specialists, and international coding and certification experts (listed under the Determination of Cause of Death [DeCoDe] panel, see Acknowledgments). The DeCoDe panel reviewed clinical, antemortem, and postmortem data to make a CoD determination, which was recorded using a modified standard reporting template (Supplementary Table 2) based on the World Health Organization’s *International Classification of Diseases*, *Tenth Revision* (*ICD-10*) for deaths during the perinatal period [11]. This included recording the “underlying condition” associated with initiating the chain of events leading or predisposing to death, subsequent antecedent medical conditions, and the final or “immediate” condition which resulted in death. The DeCoDe panel scored the level of certainty on CoD attribution for the “immediate” and “underlying” causes as level 1 (confident), level 2 (probable), and level 3 (uncertain but possible). The final CoD forms were *ICD-10* coded by a medical doctor (F. S.).

### Statistical Analysis

We stratified cases into early neonatal death (<72 hours; END) and late neonatal death (3–27 days; LND). For all variables, we calculated descriptive statistics and provided medians with interquartile range (IQR) for continuous variables and proportions for categorical variables. For select variables, differences between the END and LND were tested using χ ^2^ or Fisher exact test, with a *P* value ≤.05 considered significant. Statistical analysis was done using Stata software version 15 (StataCorp, College Station, Texas).

### Ethical Considerations

This study was approved by the Human Research Ethics Committee (reference number 150215) of the University of the Witwatersrand. Parental consent was obtained prior to any MITS procedure. Post-MITS, the study team continued to provide grief counseling.

## RESULTS

Of 236 neonatal deaths eligible for enrollment and whose parents were approached for study participation, 153 (65.7%) consented. Additional results are provided in the Supplementary Materials. There were 47 ENDs and 106 LNDs (Supplementary Figure 1). Thirty-five percent (n = 51) of neonates who died were born to HIV-infected women, including 41.5% (17/41) of ENDs and 32.5% (34/105) of LNDs (Table 1). Only 2 (1.3%) of the HIV-exposed neonates acquired HIV from the mother, diagnosed by HIV PCR. Eighty percent of cases weighed <2500 g at birth, and 78.6% were born prematurely (Table 1). The median ages on admission were 1 day and 9 days for END and LND, respectively. All of the END and 90 of the LND cases had never been discharged from hospital since birth. The median duration of hospitalization before death of the remaining 16 LNDs was 11 days (range, 3–26 days). The median time between death and undertaking the MITS was 23.4 hours (IQR, 14–37 hours) (Table 1). The majority (76.8%) of neonatal deaths with MITS had adequate core samples for histological examination, with 8.4% suboptimal samples and only 0.6% autolyzed (Supplementary Table 3).

**Table 1.** Demographic and Clinical Features of Early (<72 Hours) and Late (3–27 Days) Neonatal Deaths Investigated by Minimally Invasive Tissue Sampling

Features	Total (N = 153)	END (n = 47)	LND (n = 106)
Median (IQR) age, d, on admission	5.0 (2–11)	1.0 (0–2)	9.0 (5–14)
Male sex, No. (%)	76 (49.7)	23 (48.9)	53 (50.0)
HIV exposed	51/146 (34.9)	17/41 (41.5)	34/105 (32.4)
HIV PCR reactive^a^	2/149 (1.3)	1/47 (2.1)	1/102 (1.0)
Median (IQR) weight on admission, g	1280 (943–2410)	1485 (960–2490)	1250 (930–2395)
LBW (<2500–1500 g)	34/152 (22.4)	10/47 (21.3)	24/105 (22.9)
Very LBW (<1000–1499 g)	36/152 (23.7)	8/47 (17.0)	28/105 (26.7)
Extremely LBW (<1000 g)	51/152 (33.6)	16/47 (34.0)	35/105 (33.3)
Median (IQR) gestational age, wk	30.0 (27–36)	31 (27–37)	30 (27–35)
34 to <37 wk GA	17/140 (12.1)	5/45 (11.1)	12/95 (12.6)
28 to <34 wk GA	53/140 (37.9)	15/45 (34.1)	38/95 (39.6)
<28 wk GA	40/140 (28.6)	12/45 (26.7)	28/95 (29.5)
Significant congenital abnormalities^b^	12/114 (10.5)	4/31 (12.9)	8/83 (9.6)
Median (IQR) No. of days between admission and death	4.0 (2.0–9.0)	1.0 (0.0–2.0)	7.0 (4.0–13.0)
Median (IQR) time between death and MITS, h	23.4 (14.3–37.3)	21.6 (14.4–1.7)	24.7 (13.5–38.9)

Data are presented as no./No. (%) unless otherwise indicated.

Abbreviations: END, early neonatal death; GA, gestational age; HIV, human immunodeficiency virus; IQR, interquartile range; LBW, low birth weight; LND, late neonatal death; MITS, minimally invasive tissue sampling; PCR, polymerase chain reaction.

^a^HIV PCR test done on postmortem sample.

^b^Significant congenital abnormalities recorded in medical notes among END were 1 each of anencephaly, hydrocephalus, microcephaly, and unspecified dysmorphism; and in LND 1 each of patent ductus arteriosus, atrial septal defect, and tracheoesophageal fistula, as well as 2 hydrops fetalis and 3 exomphalos cases.

### Underlying CoD Attribution

The DeCoDe panel assigned an underlying and/or immediate CoD for all cases, except 1 tenuously attributed to “sepsis” at level 3 certainty. The most common underlying CoD categories were “low birth weight [LBW]/prematurity complications” (81/153 [52.9%]), “complications of intrapartum events” (23/153 [15.0%]), “congenital malformations” (20/153 [13.1%]), and “infection related” (15/153 [9.8%]) (Table 2). “LBW/prematurity complications” was more common as an underlying cause in LND (63/106 [59.4%]) than END (18/47 [38.3%]; *P* = .022), whereas “complications of intrapartum events” was more common in END than in LND (16/47 [34.0%] vs 7/106 [6.6%]; *P* < .001; Table 2).

**Table 2.** Underlying Cause of Death Categories and Specific Immediate Cause of Death Attribution of Early (<72 Hours) and Late (3–28 Days) Neonatal Deaths by the Determination of Causes of Death Panel

Underlying and Specific Immediate Cause of Death	Total (N = 153)	Early Neonatal Death (n = 47)	Late Neonatal Death (n = 106)	*P* Value^a^
Low birth weight/prematurity complications (N9)^a,b^	n = 81 (52.9)	n = 18 (38.3)	n = 63 (59.4)	.022
Sepsis (all)^c^	30 (37.0)	4 (22.2)	26 (41.3)	
Nosocomial sepsis^c^	26 (32.1)	2 (11.1)	24 (38.1)	
Pneumonia (all)	23 (28.4)	2 (11.1)	21 (33.3)	
Nosocomial pneumonia	21 (25.9)	1 (5.6)	20 (31.7)	
Pulmonary mucormycosis	1 (1.2)	0 (0.0)	1 (1.6)	
Meningitis (nosocomial)^d^	4 (4.9)	0 (0.0)	4 (6.3)	
Birth asphyxia^e^	1 (1.2)	1 (5.6)	0 (0.0)	
Hyaline membrane disease^f^	14 (17.3)	9 (50.0)	5 (7.9)	
Pneumothorax	1 (1.2)	1 (5.6)	0 (0.0)	
Pulmonary hemorrhage	1 (1.2)	0 (0.0)	1 (1.6)	
Intraventricular hemorrhage	4 (4.9)	1 (5.6)	3 (4.8)	
Necrotizing enterocolitis	1 (1.2)	0 (0.0)	1 (1.6)	
Hypoxic ischemic encephalopathy	1 (1.2)	0 (0.0)	1 (1.6)	
Complications of intrapartum events (N4)^a^	n = 23 (15.0)	n = 16 (34.0)	n = 7 (6.6)	< .001
Sepsis	1 (4.3)	1 (6.3)	0 (0.0)	
Nosocomial pneumonia	1 (4.3)	0 (0.0)	1 (14.3)	
Intrauterine hypoxia	4 (17.4)	4 (25.0)	0 (0.0)	
Birth asphyxia	1 (4.3)	0 (0.0)	1 (14.3)	
Hypoxic ischemic encephalopathy^g^	16 (69.6)	11 (68.8)	5 (71.4)	
Congenital malformations, deformations, and chromosomal disorder (N1)^a^	n = 20 (13.1)	n = 8 (17.0)	n = 12 (11.3)	.44
Sepsis (all)	5 (25.0)	1 (12.5)	4 (33.3)	
Nosocomial sepsis	4 (20.0)	0 (0.0)	4 (33.3)	
Pneumonia (all)	4 (20.0)	0 (0.0)	4 (33.3)	
Nosocomial pneumonia	2 (10.0)	0 (0.0)	2 (16.7)	
Vascular disorder of intestines	1 (5.0)	0 (0.0)	1 (8.3)	
Acute kidney failure	1 (5.0)	0 (0.0)	1 (8.3)	
Birth asphyxia	1 (5.0)	1 (12.5)	0 (0.0)	
Persistent fetal circulation	1 (5.0)	1 (12.5)	0 (0.0)	
Congenital malformations^h^	7 (35.0)	5 (62.5)	2 (16.7)	
Infection (N6)^a,i^	n = 15 (9.8)	n = 4 (8.5)	n = 11 (10.4)	.99
Sepsis (all)	6 (40.0)	2 (50.0)	4 (36.4)	
Nosocomial sepsis	2 (13.3)	0 (0.0)	2 (18.2)	
Pneumonia (all)^j^	3 (20.0)	0 (0.0)	3 (27.3)	
Nosocomial pneumonia	2 (13.3)	0 (0.0)	2 (18.2)	
Meningitis	3 (20.0)	0 (0.0)	3 (30.0)	
Intrauterine hypoxia	2 (13.3)	2 (50.0)	0 (0.0)	
Acquired hydrocephalus	1 (6.7)	0 (0.0)	1 (9.1)	
Respiratory and cardiovascular disorders (N7)^a^	n = 5 (3.3)	n = 1 (2.1)	n = 4 (3.8)	.99
Nosocomial pneumonia	1 (20)	0 (0.0)	1 (25.0)	
Nosocomial meningitis^k^	1 (20)	0 (0.0)	1 (25.0)	
Persistent fetal circulation^l^	3 (60.0)	1 (100)	2 (50.0)	
Convulsions and disorders of cerebral status (N5)^a^	n = 1 (0.7)	n = 0	n = 1 (0.9)	.99
Nosocomial pneumonia^m^	1 (100)	0 (0.0)	1 (100)	
Other neonatal conditions (N8)^a^	n = 7 (4.6)	n = 0	n = 7 (6.6)	.10
Subarachnoid hemorrhage	1 (14.3)	0 (0.0)	1 (14.3)	
Candidiasis	1 (14.3)	0 (0.0)	1 (14.3)	
Kernicterus due to isoimmunization	3 (42.9)	0 (0.0)	3 (42.9)	
Necrotizing enterocolitis	1 (14.3)	0 (0.0)	1 (14.3)	
Hypoxic ischemic encephalopathy	1 (14.3)	0 (0.0)	1 (14.3)	
Neonatal death of unspecified cause (N11)^a^	n = 1 (0.7)	n = 0 (0.0)	n = 1 (0.9)	.99
Shock	1 (100)	0 (0.0)	1 (100)	

Data are presented as No. (%) unless otherwise indicated. Causes of death (CoD) are according to the World Health Organization *International Classification of Diseases, Tenth Revision* (*ICD-10*) application to perinatal mortality.

^a^Indicates *ICD-10* code categories.

^b^Comparisons of immediate CoD not done due to limited power.

^c^Four cases had pneumonia as coimmediate CoD and are thus counted under pneumonia. Two of the cases had the same pathogen for sepsis and pneumonia, whereas the other 2 had different pathogens causing the sepsis and pneumonia.

^d^Two nosocomial *Acinetobacter baumannii* meningitis cases had nosocomial pneumonia as coimmediate CoD; 1 was *A. baumannii* and the other was *Klebsiella pneumoniae.*

^e^This case of birth asphyxia had hyaline membrane disease (HMD) as coimmediate CoD.

^f^Two cases of HMD had other coimmediate CoD. One had persistent fetal circulation and the other had unspecified pneumonia.

^g^One case had HMD and another case had meconium aspiration syndrome as coimmediate CoD to hypoxic ischemic encephalopathy.

^h^One case had 2 coimmediate CoD, both congenital malformations: congenital hypoplasia and dysplasia of lung and Potter syndrome.

^i^Six cases had the same pathogen as the immediate and underlying CoD.

^j^One case of pneumocystis pneumonia had rotavirus gastroenteritis as a coimmediate CoD.

^k^The case of nosocomial *A. baumannii* meningitis had nosocomial *A. baumannii* pneumonia as a coimmediate CoD.

^l^One case of persistent fetal circulation also had nosocomial *A. baumannii* sepsis as a coimmediate CoD.

^m^This case of nosocomial *K. pneumoniae* pneumonia also had nosocomial sepsis (*Enterobacter cloacae and A. baumannii*) as coimmediate CoD.

Overall, the DeCoDe panel scored their diagnoses on the underlying CoD as level 1, level 2, and level 3 certainty in 88.2%, 9.8%, and 2% of cases, respectively (Supplementary Table 4*A*). Similarly, there was a high level of confidence in attributing an immediate CoD (89.5%), whereas 9.2% and 1.3% of the immediate CoD diagnoses were considered as level 2 or level 3, respectively (Supplementary Table 4*B*).

### Immediate CoD in Cases With an Underlying Diagnosis of LBW/Prematurity Complications

Among deaths attributed to “LBW/prematurity complications” as the underlying cause, infections were the immediate CoD in 70.4% (58/81) of cases; more commonly so in LND (52/63 [82.5%]) than among END (6/18 [33.3%]; *P* < .001) (Table 2). The specific infection-related diagnoses included sepsis (30/81 [37.0%]), pneumonia (23/81 [28.4%]), and meningitis (4/81 [4.9%]), of which 86.7% (26/30), 91.3% (21/23), and 100% (4/4), respectively, were hospital acquired. Hospital-acquired sepsis, pneumonia, and meningitis were more common as an immediate CoD among LND (48/63 [76.2%]) than END (3/18 [16.6%]; *P* < .001) in cases with “LBW/prematurity complications” as the underlying cause. Histologically confirmed hyaline membrane disease was more common as the immediate cause among ENDs (9/18 [50.0%]) than LNDs (5/63 [7.9%]) (*P* < .001).

### Immediate CoD in Cases With Underlying Diagnoses Other Than LBW/Prematurity Complications

Among deaths attributed to “congenital malformations” as the underlying cause, infections were the dominant (9/20 [45%]) immediate CoD, two-thirds of which were hospital acquired, whereas 35% (7/20) of these deaths were a direct consequence of the underlying congenital abnormality (Table 2).

Deaths attributed to “complications of intrapartum events” were mainly due to hypoxic brain damage, 71.4% (15/21) of which were END cases. Hypoxic brain injury was also attributed as an immediate CoD in neonates with underlying causes other than “complications of intrapartum events,” with an overall prevalence of 17.0% (26/153); and more commonly so among END (18/47 [38.3%]) than LND (8/106 [7.5%]) (*P* < .001; Table 2).

### Infectious Diseases as an Underlying or Immediate CoD

**Figure 1.**
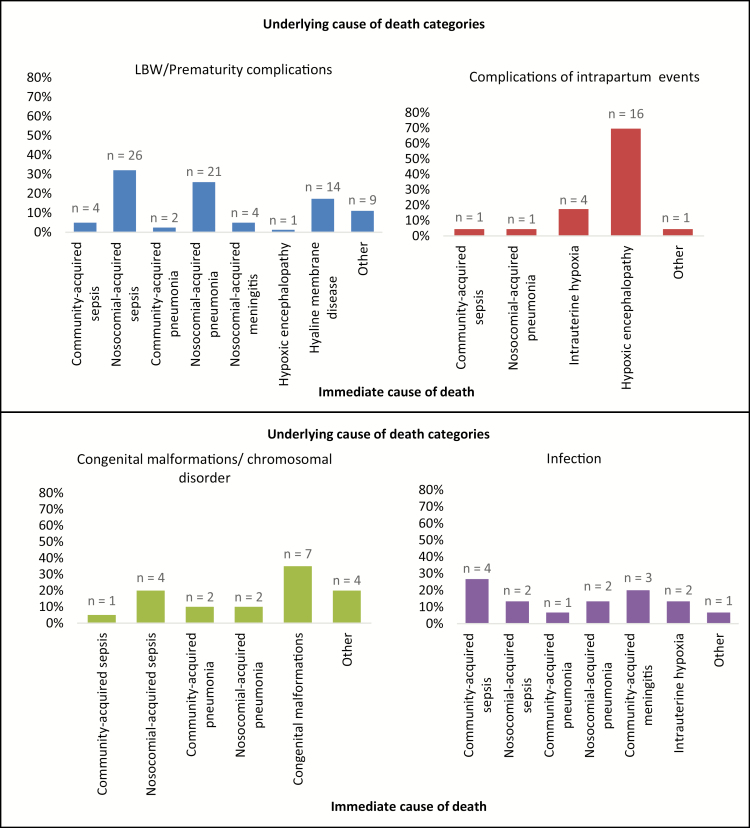
Immediate causes of death by leading underlying cause of death category in all neonates. “Other” includes persistent fetal circulation, intraventricular hemorrhage, birth asphyxia, kernicterus, necrotizing enterocolitis, pulmonary mucormycosis, vascular disorders of intestines, subarachnoid hemorrhage, acute kidney failure, pneumothorax, pulmonary hemorrhage, shock, and acquired hydrocephalus. Abbreviation: LBW, low birth weight.

Overall, community-associated infections were the underlying and/or immediate CoD in 15.0% (23/153) of cases, including sepsis (9.2%), pneumonia (5.9%), and meningitis (2.0%) (Table 3). The leading pathogens identified among the 23 community-associated infection–related deaths included group B *Streptococcus* (GBS) (n = 5 [21.7%]), *Escherichia coli* (n = 4 [17.4%]), and *Staphylococcus aureus* (n = 2 [8.7%]). Among the 15 deaths with “infections” as the underlying CoD, 5 (33.3%) were due to GBS (Table 3). Furthermore, 8 of these 15 cases died directly from the initial infection, and 4 died from a subsequent hospital-acquired infection (Figure 1).

**Table 3.** Pathogens Identified in Early (<72 Hours) and Late (3–27) Neonatal Deaths in Which the Immediate or Underlying Cause of Death Was Attributed to Sepsis, Pneumonia, or Meningitis

Diagnosis and Pathogen	Nosocomial Infections				Community-Acquired Infections				Overall Total
	Sepsis	Pneumonia	Meningitis	Total	Sepsis	Pneumonia	Meningitis	Total	
Total for column	n = 38 (24.8)^a^	n = 32 (20.9)^b, c^	n = 5 (3.3)^d^	67 (43.8)^e, f, g^	n = 14 (9.2)^h^	n = 9 (5.9)	n = 3 (2.0)	n = 23 (15.0)^h,i^	n = 90 (58.8)^j^
*Acinetobacter baumannii*	18 (47.4)	17 (53.1)	5 (100)	35^g,k^ (52.2)	0	0	0	0	35 (38.9)
*Klebsiella pneumoniae*	6 (15.8)	10 (31.3)	0	15^l^ (22.4)	0	1 (11.1)	0	1 (4.3)	16 (17.8)
*Escherichia coli*	4 (10.5)	1 (3.1)	0	4^m^ (6.0)	4^n^ (28.6)	0	1^n^ (33.3)	4 (17.4)	8 (8.9)
*Staphylococcus aureus*	9 (23.7)	5 (15.6)	0	14^g^ (20.9)	2 (14.2)	0	0	2 (8.7)	16 (17.8)
*Enterococcus faecium/faecalis*	3 (7.9)	2 (6.3)	0	5 (7.5)	1 (7.1)	0	0	1 (4.3)	6 (6.7)
Group B *Streptococcus*	0	0	0	0	4^o^ (28.61)	1 (11.1)	2^o^ (66.7)	5 (21.7)	5 (5.6)
*Candida parapsilosis*	3 (7.9)	0	0	3 (4.5)	0	0	0	0	3 (3.3)
*Ureaplasma* spp	1 (2.6)	1 (3.1)	0	2 (3.0)	0	1 (11.1)	0	1 (4.3)	3 (3.3)
Cytomegalovirus^p^	0	0	0	0	0	2 (22.2)	0	2 (8.7)	2 (2.2)
Coagulase-negative *Staphylococcus*	2 (5.3)	0	0	2 (3.0)	0	0	0	0	2 (2.2)
Respiratory syncytial virus	0	1 (3.1)	0	1 (1.5)	0	1 (11.1)	0	1 (4.3)	2 (2.2)
*Enterobacter cloacae*	1 (2.6)	0	0	1 (1.5)	0	0 (0.0)	0	0	1 (1.1)
*Serratia marcescens*	1 (2.6)	0	0	1 (1.5)	0	0 (0.0)	0	0	1 (1.1)
*Haemophilus influenzae*	0	0	0	0	0	1 (11.1)	0	1 (4.3)	1 (1.1)
Influenza virus	0	0	0	0	0 (0.0)	1 (11.1)	0	1 (4.3)	1 (1.1)
*Candida albicans*	0	0	0	0	1 (7.1)	0	0	1 (4.3)	1 (1.1)
*Stenotrophomonas maltophilia*	0	1 (3.1)	0	1 (1.5)	0	0	0	0	1 (1.1)
*Pseudomonas aeruginosa*	0	1 (3.1)	0	1 (1.5)	0	0	0	0	1 (1.1)
*Pneumocystis jirovecii*	0	0	0	0	0	1 (11.1)	0	1 (4.3)	1 (1.1)
Unspecified infection	0	0	0	0	3 (21.4)	0	0	3 (13.0)	3 (3.3)

Data are presented as No. (%).

^a^Total; number is less than the number of listed organisms because of the following 9 coinfections: *E. faecium* and *K. pneumoniae*; *E. coli* and *K. pneumoniae*; *E. coli* and *S. aureus*; *Enterobacter* and *K. pneumoniae*; *A. baumannii* and *E. coli*; *A. baumannii* and *S. aureus* (×2); *A. baumannii*, *S. aureus*, and *Ureaplasma*; *A. baumannii*, *E. faecium*, and *S. aureus*.

^b^Five LND cases had both pneumonia and sepsis: *K. pneumoniae* pneumonia and *Enterobacter* spp and *K. pneumoniae* sepsis; methicillin-resistant *S. aureus* (MRSA) pneumonia and *A. baumannii* sepsis; both sepsis and pneumonia from *A. baumannii* and *E. coli*; *K. pneumoniae* sepsis and *A. baumannii* pneumonia; both sepsis and pneumonia from *A. baumannii*.

^c^No. is less than the total number of listed organisms because of the following 7 coinfections: *E. faecalis* and *A. baumannii*; respiratory syncytial virus and *A. baumannii*; *A. baumannii*, *Ureaplasma*, and *K. pneumoniae*; *A. baumannii* and *E. coli*; *A. baumannii* and *K. pneumoniae*; *K. pneumoniae* and *Enterococcus* species; MRSA and *K. pneumoniae.*

^d^Three had coinfections with nosocomial pneumonia (*A. baumannii* meningitis with *A. baumannii* pneumonia [n = 2] and *A. baumannii* meningitis with *K. pneumoniae* pneumonia [n = 1]), and all were late neonatal deaths.

^e^The row total is less (n = 8), which are the cases that had both pneumonia and sepsis (n = 5) and those that had pneumonia and meningitis (n = 3).

^f^No. is less than the total of organisms listed below because of the coinfections indicated by footnotes b and d.

^g^Only 3 were early neonatal deaths from *A. baumannii* sepsis (n = 2) and *S. aureus* pneumonia (n = 1), whereas 64 were late neonatal deaths.

^h^No. is less than the total number of listed organisms because of 1 case with *E. faecalis* and *S. aureus* coinfection.

^i^The row total is less (n = 3), where *E. coli* was the pathogen for both sepsis and meningitis (n = 1, see footnote n) and GBS was the pathogen for both sepsis and meningitis (n = 2, see footnote o).

^j^No. is less than the total of organisms listed below because of the coinfections between indicated by footnotes e and h.

^k^The row total is less (n = 5), which are the cases in which *A. baumannii* was the pathogen for both pneumonia and sepsis (n = 2) as well as pneumonia and meningitis (n = 3).

^l^The row total is less (n = 1), which are the cases in which *K. pneumoniae* was the pathogen for both pneumonia and sepsis (n = 1).

^m^The row total is less (n = 1), which are the cases in which *E. coli* was the pathogen for both pneumonia and sepsis (n = 1).

^n^The *E. coli* meningitis (immediate) case had *E. coli* sepsis as underlying cause of death.

^o^The 2 group B *Streptococcus* (GBS) meningitis (immediate cause of death) cases had underlying GBS sepsis.

^p^One case of cytomegalovirus had coinfection with nosocomial MRSA pneumonia.

In 67 (74.4%) deaths with “infections” as either an underlying or immediate CoD (n = 90), the infections were hospital-acquired, including 5 with concurrently diagnosed sepsis and pneumonia caused by different pathogens in the blood and lung as the immediate CoD (Table 3). Hospital-acquired infections were more common in LND (64/106 [60.4%]) than END (3/47 [6.4%]) (*P* < .001; Table 2). The most common hospital-acquired pathogens were *Acinetobacter baumannii* (52.2%), *Klebsiella pneumoniae* (22.4%), *S. aureus* (20.9%), *E. coli* (6.0%), *Enterococcus faecium* (7.5%), and *Candida parapsilosis* (4.5%) (Table 3).

The antibiotic resistance profile of bacteria implicated as an underlying or immediate CoD is tabulated in Supplementary Table 5. *Acinetobacter baumannii* isolates were generally resistant to all classes of antibiotics, including 100% (46/46) to carbapenems and 100% (45/45) to piperacillin/tazobactam, but sensitive to colistin. Similarly, *K. pneumoniae* isolates were generally resistant to most classes of antibiotics, including 73.9% (17/23) to cefotaxime, but sensitive to carbapenems and amikacin (4.0% [1/25]) resistance); albeit generally resistant to other aminoglycosides. One hundred percent (15/15) of *S. aureus* isolates were methicillin resistant.

## DISCUSSION

This study demonstrated that MITS, coupled with medical record review, is a robust method to determine the underlying and highly specific immediate causes of deaths occurring in neonates in a LMIC setting. The acceptability of MITS in our study, where two-thirds of bereaved parents agreed to study participation, was similar to the hypothetical acceptability thereof in a multicenter study [8] and affirms its ability to bridge the data gap arising from inter alia cultural and religious barriers in undertaking CDA in LMICs [8].

The data generated from this pilot study to the CHAMPS program provide proof of principle that MITS is feasible with informative findings. These findings set the stage for multicenter mortality surveillance, which could be instrumental in prioritizing strategies and interventions to reduce neonatal mortality [6]. This could include identifying and addressing diseases, which could be prevented, or treated in neonatal deaths attributed to underlying conditions such as “LBW/preterm complications,” which need not be life-threatening. An overwhelming finding from our study was the dominant but underemphasized role of hospital-acquired infections to in-facility neonatal deaths, especially in LNDs (60.4%). Notably, the dominant pathogen, *A. baumannii*, was resistant to all classes of antibiotics except for colistin, which is not licensed for use in neonates in South Africa. The observations on the contribution of hospital-acquired infections as a cause of neonatal deaths in this study have underpinned a reevaluation and introduction of strategies aimed to improve infection control practices at CHBAH, the success of which will be evaluated through the ongoing CHAMPS program in Soweto.

Our study also demonstrates the need to evaluate cause-specific mortality fraction beyond analysis of the underlying CoD as is currently the focus of estimates on causes of neonatal deaths [2]. Notably, infections that could potentially be prevented or treated were the immediate CoD among 70.4% and 45.0% of cases with “LBW/prematurity complications” and “congenital abnormalities,” respectively, which were among the 3 leading underlying CoD. Furthermore, 79.8% of LNDs were infection related—also almost exclusively due to hospital-acquired infections. In contrast, among ENDs with “LBW/prematurity complications” as an underlying CoD, the immediate CoD were predominantly due to histologically confirmed hyaline membrane disease (50%) and other common complications of prematurity.

The value of postmortem bacterial culture in attributing a casual association to death is controversial due to multiple potential sources of sample contamination, including postmortem aspiration of gastrointestinal and upper airway colonizing bacteria, or translocation of enteric bacteria into the bloodstream. Nevertheless, implicating bacterial infection as the CoD in this study was done only after review of each individual case, and considering multiple corroborating lines of evidence available for each death. For example, pneumonia-attributed deaths were corroborated by lung pathology, showing histological evidence of pneumonia, coupled with immunohistochemical evidence of infection, in addition to PCR detection and/or culture positivity. Also, sepsis was typically diagnosed based on culture and/or PCR positivity from >1 site (postmortem) if premortem culture was negative or PCR positivity from at least 1 site (postmortem) coupled with premortem culture showing the same organism, and often with histopathological evidence of sepsis from multiple organs. Furthermore, in support that MITS sampling for identifying infectious-related deaths was not attributable to environmental contamination was the difference in frequency of identification and spectrum of pathogens implicated as the cause of infection between END and LND cases. The dominant bacteria implicated as causing infection-related END were those commonly colonizing the maternal rectovaginal tract in our setting [12], which could cause invasive disease in the newborn following bacterial infection in utero or during birth. Nevertheless, the possibility of postmortem contamination by these organisms cannot be excluded.

Although the burden of hospital infections and spectra of implicated pathogens might be site-specific [13], the importance of hospital infection as a cause of neonatal mortality has also been observed in other LMIC settings on antemortem sampling. In Zambia, high case fatality ratios (29%–47%) were reported in hospitalized neonates with suspected sepsis, among whom extended-spectrum β-lactamase *K. pneumoniae* was the dominant (75%) pathogen and primarily hospital-acquired [14]. Gram-negative bacteria, particularly *Acinetobacter* species (22%) and *Klebsiella* species (17%), were also the dominant cause of neonatal sepsis in India, with high rates of multidrug resistance observed for *Acinetobacter* species (82%) and *Klebsiella* species (54%). Notably, one-quarter of neonatal deaths in the Indian study were attributable to sepsis based on antemortem sampling [15], which could be an underestimate due to low-to-moderate sensitivity of blood culture in detecting bacterial infections [13].

The proportional distribution of underlying CoD in neonates in our study differed from that estimated nationally for South Africa in 2015 [2]. These differences included a higher percentage of deaths in our study being attributed to “LBW/prematurity complications” (52.9% vs 35%) and “congenital abnormalities” (9.2% vs 3.1%), and a lower percentage to “complications of intrapartum events” (15.0% vs 21.4%), and “infections” (9.8% vs 20.6%). The national estimates are, however, based on passive reporting from vital registration databases and limited verbal autopsy reports, which could explain the discordance. A limitation of our study, and the comparison to the national estimates, was that it was a single facility-based study; hence, our findings may not be generalizable to the broader community or other settings with different levels of healthcare access. Although a strength of the study setting was the presence of a single public referral hospital in Soweto, we could have missed community deaths not brought to the hospital.

Another study limitation was that the commercially available multiplex PCR assay was not customized for this study, including lacking targets to evaluate for the most common bacteria (ie, *A. baumannii* and *K. pneumoniae*) causing hospital-acquired infections.

Notably, the distribution of timing of neonatal deaths represented in our study differs from the global trend, where approximately 50% of neonatal deaths are estimated to occur within 72 hours of birth [16], compared to 30.7% of cases sampled in our study. This could be explained by the majority (>95%) of deliveries in our setting occurring within healthcare facilities, as well as reasonable access to curative healthcare including intensive care facilities, and hence a better chance of neonatal survival within 72 hours of birth. Furthermore, the exclusion of newborns with birth weight of <750 g, who are highly likely to die within a few hours of birth in the absence of full access to intensive care interventions, including in our setting, might have also contributed to these differences.

The aspiration of providing “universal healthcare” as envisioned in the United Nations Sustainable Development Goal 3.8 [17], could possibly lead to epidemiological shifts in the timing of neonatal deaths globally. This includes potentially a greater role of hospital-acquired infections as an immediate cause of neonatal deaths, as the number of deliveries attended by skilled healthcare workers has steadily increased from 60% in 2000 to 80% by 2015 globally [18], and many babies born preterm are now more likely to stay in hospital after delivery. Consequently, greater focus may be required now to mitigate the risk of hospital infections, particularly in prematurely born neonates, as alluded to in our study.

In conclusion, in this proof-of-concept study to the CHAMPS program, we demonstrate the potential role of MITS interpreted with other clinical information in addressing the knowledge gap on causes of neonatal mortality. Future surveillance using MITS in the multicenter CHAMPS program will contribute to inform decision making in the prioritization of interventions and research that need to be undertaken to reduce neonatal deaths.

## Supplementary Data

Supplementary materials are available at *Clinical Infectious Diseases* online. Consisting of data provided by the authors to benefit the reader, the posted materials are not copyedited and are the sole responsibility of the authors, so questions or comments should be addressed to the corresponding author.

ciz574_suppl_Supplementary_Material

ciz574_suppl_Supplementary_Figure-1

## Notes

***Author contributions.*** Conceptualization and methodology, supervision, funding acquisition, writing of the first draft: S. A. M. Data curation: S. A. M., R. C., J. P., V. B., A. I., S. N., F. S., A. M., M. H., S. Z., J. W., F. L. N. Data analysis: S. A. M., R. C., A. I. Laboratory testing: V. B., M. H., S. Z., J. W. Project administration: A. M., S. N., R. C. Training of staff on minimally invasive tissue sampling: Q. B., J. O. Manuscript review and editing: S. A. M., R. C., J. P., V. B., A. I., S. N., F. S., A. M., M. H., S. Z., J. W., F. L. N., S. V., Q. B., J. O.

***Acknowledgments.*** The authors acknowledge the following pathologists for their contribution to undertaking diagnostic histopathology investigations: Dr S. Pather, Dr R. Mohanlal, Dr E. van den Berg, Ms Marie Suleman, and Dr R. Wadee (all from the National Health Laboratory Service Department of Anatomical Pathology, Faculty of Health Sciences, University of the Witwatersrand). The authors acknowledge the important contributions of the Determining Cause of Death (DeCoDe) panel (speciality in parenthesis) that was chaired by Scott Dowell (epidemiologist, pediatrician) and Christopher Wilson (infectious diseases, pediatrician). Other DeCoDe panel members were Quique Bassat (infectious diseases, pediatrician), Dianna Blau (microbiology), Robert Breiman (epidemiology, infectious diseases, physician), Daniel Chandramohan (epidemiology, World Health Organization *International Classification of Diseases* coding specialist), Richard Chawana (physician), Kevin Clarke (microbiology), Martin Hale (pathology), Penny Heaton (physician), Eric Houpt (microbiology, infectious disease, physician), Aggrey Igunza (study clinician), Grace Irimu (pediatrician), Reinhard Kaiser (epidemiology, physician), Nicholas Kassebaum (microbiology), Keith Klugman (microbiology, infectious diseases, physician), Karen Kotloff (infectious diseases, pediatrician), Shabir Madhi (infectious diseases, pediatrician), Kim Mulholland (infectious diseases, epidemiology, pediatrician), Jaume Ordi (pathology), Robert Pattinson (obstetrician), Rebecca Philipsborn (pediatrician), Pratima Raghunathan (epidemiology), Anna Seale (infectious diseases, pediatrician), Milagritos Tapia (infectious diseases, pediatrician), Allan Taylor (epidemiology, pediatrician), and Sherif Zaki (pathology).

***Disclaimer.*** The findings and conclusions in this report are those of the author(s) and do not necessarily represent the official position of the Centers for Disease Control and Prevention. This work was funded by the Bill & Melinda Gates Foundation (BMGF), who played no role in the study design, data collection and analyses, preparation, or approval of this manuscript. BMGF staff participated in the DeCoDe panel, and the program officer (Scott Dowell) and Chris Wilson were co-chairs of the DeCoDe panel and were provided the opportunity to review a preliminary version of this manuscript for factual accuracy, but the authors are solely responsible for final content and interpretation.

***Financial support.*** This work was supported by the Bill & Melinda Gates Foundation [OPP1101764].

***Supplement sponsorship.*** This supplement is sponsored by the Emory Global Health Institute and the Bill & Melinda Gates Foundation.

***Potential conflicts of interest.*** S. A. M. has received grants from the Bill & Melinda Gates Foundation, Pfizer, Glaxo Smith Kline, Biovac, Novavax, and Sanofi Pasteur; and honoraria from The Bill & Melinda Gates Foundation. All other authors report no potential conflicts of interest. All authors have submitted the ICMJE Form for Disclosure of Potential Conflicts of Interest. Conflicts that the editors consider relevant to the content of the manuscript have been disclosed.
