# Supplementary material for: Unraveling Specific Causes of Neonatal Mortality Using Minimally Invasive Tissue Sampling: An Observational Study
Source: Clin Infect Dis. 2019 Oct 9;69(Suppl 4):S351–60. doi: 10.1093/cid/ciz574 (PMC6785687; doi:10.1093/cid/ciz574)
Supplement: ciz574_suppl_Supplementary_Material [file ciz574_suppl_supplementary_material.docx]

**Supplementary material: Unravelling specific causes of neonatal mortality using minimal invasive tissue sampling: An observational pilot study.**

**Methods supplement**

***Study site and population***

Although categorized as a middle income country, wealth distribution is highly disparate in South Africa, with 19% of households surviving on less than $2 per day. The majority of Black Africans are among the lowest income categories, including the majority of the 40% unemployed workforce. Soweto is the largest urban settlement in South Africa, constituted almost exclusively of Black Africans, with a high unemployment rate but reasonable access to public health facilities. The population of Soweto is estimated at 1.2 million, including an under-5 population of 128,000 and annual birth cohort of 28,000. The majority (99%) of deliveries in Soweto occur at public health facilities, where health care is provided at no cost to all pregnant women and children by the State. Three quarters of all births in the public health sector in Soweto occur at Chris Hani Baragwanath Academic Hospital (CHBAH) and the others at one of 7 midwife operated units (MOUs). At the time of the study, CHBAH was the only public hospital in Soweto.

Furthermore, there is a low threshold for referrals from the MOUs to the hospital if any signs of imminent obstetric complications are observed before or during labour, including women in preterm labour . Also, there is a low threshold for referring ill neonates from the surrounding primary health care clinics to CHBAH for further management. Facilities at CHBAH include a neonatal intensive care unit where invasive and non-invasive mechanical ventilator support is available. Due to resource constrains, the policy at CHBAH is to limit invasive mechanical ventilatory support to newborns weighing at least 1,000 grams at birth, whilst non-invasive mechanical ventilation such as continuous positive airway pressure is available to those weighing <1,000 grams if clinically indicated.

The prevalence of HIV among pregnant women in Soweto has remained unchanged at 28-29% over the past decade, however, improved mother-to-child HIV prevention strategies have resulted in HIV transmission rates to HIV-exposed infants declining from 8% in 2004 to 1.1% by 2015 [1]. The neonatal mortality rate in Soweto, based on a longitudinal cohort study from 2011 was estimated to be 22 per 1000 live births (unpublished data from Matflu study) [2], which was higher than the national rate of 14 per 1000 live births estimated for South Africa in 2015 [3].

***Minimal invasive tissue sampling (MITS) procedures:***

The MITS was undertaken either by a medical doctor or professional nurse assisted by research assistants. Study staff observed standardised procedures, including use of personal protective equipment, and other precautionary measures to avoid contamination of samples. The corpse was first washed using water, then disinfected using 70% ethanol to mitigate contamination of the samples obtained.

Using the Bard Monopty 14-gauge disposable core biopsy needles (Bard Peripheral Vascular Inc, Temple, USA), the chest wall was punctured through the 6^th^ intercostal space posteriorly and angling the needle towards the different lung zones. Lung tissue was collected from the upper, middle and lower zones. The left and the right lung tissue samples were processed separately. Liver tissue from the left and right lobes was collected through a puncture in the 10^th^ intercostal space mid-axillary line and angled towards the xiphoid process to reach the left lobe. Brain tissue was collected using the Bard Max-core 18-gauge disposable biopsy needle (Bard Peripheral Vascular Inc, Temple, USA) through either a transnasal approach (through the cribriform plate of the roof of the nose) or through the anterior fontanelle.

Tissue samples for culture were placed in sterile normal saline containing jars, and those for histology in 10% neutral buffered formalin containing jars. In all instances, the first tissue biopsy sample was sent for culture, the second for molecular test (lung only) and subsequent six core samples obtained were sent for histopathology examination.

***Bacterial culture and antibiotic susceptibility testing methods***

Blood was collected following decontamination of the skin surface using alcohol solution and through cardiac puncture or by supraclavicular approach into the left subclavian vessel and was placed into an EDTA tube and then 0.5-5 ml inoculated into Bact/ALERT PF Plus bottle and evaluated using the BacT/Alert microbial system (BioMerieux, Marcy l’Etoile, France) at the National Health Laboratory Services (NHLS) at CHBAH. All positive cultures from the various sample types were Gram stained and further identified using standard manual methods. Cerebrospinal fluid was collected through a puncture into the cisterna magna posteriorly and placed in sterile tube and cultured using culture plates. Gram staining, culture identification and antibiotic susceptibility testing were done according to clinical laboratory standard institute (CLSI) guidelines following culture.

***Molecular diagnostic testing***

Liver and lung tissues for molecular testing were collected in the Qiagen lysis buffer. The biopsies were macerated in 600µL of sterile saline and subsequently mixed with a 25mg/mL proteinase K in 20mM Tris-HCl, pH 8.3, stock solution in a 1:1 ratio. The biopsy containing solution was incubated at 55^o^C for 15 minutes and vortexed every 5 minutes.

Total nucleic acids were extracted from 400µL of the lung biopsy solution and from 200µL of the whole blood and cerebral spinal fluid samples using the NucleiSens EasyMag extraction system as per manufactures instructions (BioMerieux, Marcy l'Etoile, France) using the Specific B off-board extraction protocol. Whereas, nucleic acids were extracted from 400µL of stool samples using the NucleiSens EasyMag extraction system as per manufactures instructions (BioMerieux, Marcy l'Etoile, France) using the standard on-board extraction protocol.

The total nucleic acid specimens were evaluated with FTD multiplexed real-time PCR assays according to manufactures instructions. RT-PCR for all FTD kits were performed using the Applied Biosystems 7500® instruments (Applied Biosystems, Foster City, CA) using the following cycling conditions: 50^o^C for 15 minutes, 95^o^C for 10 minutes followed by 40 cycles of 95^o^C for 8 seconds and then 60^o^C for 34 seconds. The kits used for testing of different samples and the target organism probes included in the individual kits is outlined in Supplementary Table 1.

Each assay included template and non-template controls as well as external and internal controls supplied with the kits. A cycle threshold (Ct) cut-off above 35 Ct was implemented for all FTD kits and only those with Ct<35 were considered as “positive” provided the run passed the controls quality criteria.

***Determination of Cause of Death (DeCoDE) process***

The DeCoDE panel convened from 26^th^ March to 5^th^ April 2017 in South Africa and reviewed each individual case. Following a summarised presentation of each case by either SAM or RC, the panel under the Chair of either CW or SD attributed the CoD. All the MITS results and hospital medical records were made available to the DeCoDE panel. The CoD attribution was based on the WHO International Classification of Diseases, 10^th^ revision (ICD-10) for deaths during the perinatal period (ICD-PM) [4], using a modified standard CoD reporting template; Supplementary Table 2. This included recording the “underlying condition” considered to have initiated the chain of events which led to or predisposed to the death. Furthermore, antecedentg conditions and the ultimate event which led to death (“immediate cause”) were also evaluated for. The perinatal deaths per ICD-10 guidelines are classified in a 3-stage process: 1) Firstly, neonatal deaths are grouped according to timing (early neonatal: up to day 7 of postnatal life; or late neonatal: days 8–28 of postnatal life), 2) The main cause of perinatal death is assigned and grouped according to the ICD-PM groupings. 3. The main maternal condition at the time of perinatal death is assigned and grouped according to the ICD-PM groupings, if applicable. Maternal data were unavailable for analyses in our study.

As an example, a prematurely born neonate who died following a hospital-acquired infection, would have “prematurity and its complications” attributed as the “underlying” CoD and nosocomial sepsis as the “immediate” CoD. In contrast, a prematurely born baby dying from invasive bacterial infection on Day 0 of life, could have the underlying (and immediate) CoD attributed to the invasive bacterial disease if the infection was considered to have possibly precipitated the preterm birth. The ultimate decision regarding the underlying and immediate causes in cases that were not straightforward, was taken by consensus of the panel members after discussion of each individual case.

The DeCoDE panel agreed to listing up to two “immediate” (i.e. co-immediate) CoD if there were similar levels of evidence for different diseases having contributed to the death, including infectious causes in which the individual role of different pathogens could not be prioritised. An example of this is a two-day-old neonate with co-immediate CoD attributed to histologically confirmed cytomegalovirus (CMV) pneumonitis and nosocomial methicillin resistant *Staphylococcus aureus* (MRSA) with the underlying CoD attributed to “LBW/prematurity complications”. In the event that the panel determined a single pathogen to be the CoD, despite multiple putative pathogens being identified, the other pathogens were listed either as antecedent causes or as a possible “contributing” factors but not directly implicated in the causal pathway of the death. The final CoD forms were ICD-10 coded by a medical doctor (FS). Consensus was reached for all cases on the CoD by the DeCoDe panel.

**Results supplement**

**Supplementary table 1: Type of post-mortem specimen collected, location of testing and the type of testing undertaken on the collected tissues**

| Specimen type | Lab | Early Neonatal deaths (<72hours age) | Late Neonatal deaths  (3 -28 days age) |
| --- | --- | --- | --- |
| Blood | NHLS-Micro^1^ | MC&S^4^ | MC&S |
|  | RMPRU^2^ | FTD^5^ -Sepsis kit^6^ | FTD Sepsis kit |
| Cerebrospinal fluid | NHLS-Micro | MC&S | MC&S |
|  | RMPRU | FTD Neuro-9^7^ and Sepsis kit | FTD Neuro-9 and Sepsis kit |
| Lung tissue | NHLS-Micro | MC&S | MC&S |
|  | NHLS-Histo^3^ | Histology | Histology |
|  | RMPRU | FTD Neuro-9 and Sepsis kit | FTD Neuro-9, Sepsis and Resp-33^8^ panel kit |
| Liver tissue | NHLS-Micro | MC&S | MC&S |
|  | NHLS-Histo | Histology | Histology |
|  | RMPRU | Archive | Archive |
| Brain tissue | NHLS-Micro | MC&S | MC&S |
|  | NHLS-Histo | Histology | Histology |
|  | RMPRU | Archive | Archive |
| Rectal swab | RMPRU | FTD-Gastro kit^9^ | FTD-Gastro kit |

^1^NHLS-micro: National Health Laboratory Service microbiology department. ^2^RMPRU: Respiratory and Meningeal Pathogens Research Unit laboratory. ^3^NHLS-Histo: National Health Laboratory Service, Anatomical Pathology department. ^4^MC&S= Microscopy, culture and antibiotic susceptibility testing

^5^FTD=Fast Track Diagnostics multiple PCR kits.

^6^FTD sepsis kit PCR probes for Cytomegalovirus, Group B *streptococcus* (*Streptococcus agalactiae)*, *Listeria monocytogenes, Escherichia coli, Staphylococcus aureus, Chlamydia trachomatis* and *Ureaplasma urealyticum/parvum*

^7^FTD Neuro-9 PCR probes for: cytomegalovirus (CMV), Epstein-Barr Virus, Adenovirus, herpes simplex virus 1 and 2, varicella-zoster virus, enterovirus, parechovirus, human herpes virus 6 and 7 and parvovirus B19.

^8^FTD Resp-33 PCR panel probes for: cytomegalovirus, influenza C, influenza A, influenza B, rhinovirus, coronavirus NL63, 229E, OC43, HKU1, parainfluenza 1, 2, 3, 4, human metapneumovirus A/B, bocavirus, respiratory syncytial virus A/B (RSV), adenovirus, enterovirus, parechovirus, *Mycoplasma pneumoniae* *Chlamydia pneumoniae, Staphylococcus aureus, Streptococcus pneumoniae, Haemophilus influenza*, *Pneumocystis jirovecii, Haemophilus influenzae* type B, *Bordetella* spp. (except *Bordetella parapertussis*), *Moraxella catarrhalis, Klebsiella pneumoniae, Legionella spp.* and *Salmonella spp*.

^9^FTD-Gastro kit: Probes for Enterohemorrhagic verotoxin producing *E. coli*; Norovirus G1 and G2; Adenovirus; Astrovirus; Sapovirus; *Campylobacter; Salmonella* sp*.; Yersinia enterocolitica; Cryptosporidium* spp, Rotavirus, *Clostridium difficile*, *Shigella/Enteroinvasive E.coli, Entamoeba histolytica, Giardia lamblia*

**Supplementary Table 2: Template of cause of death form completed by the Determination of Cause of Death (DeCoDe) panel**.


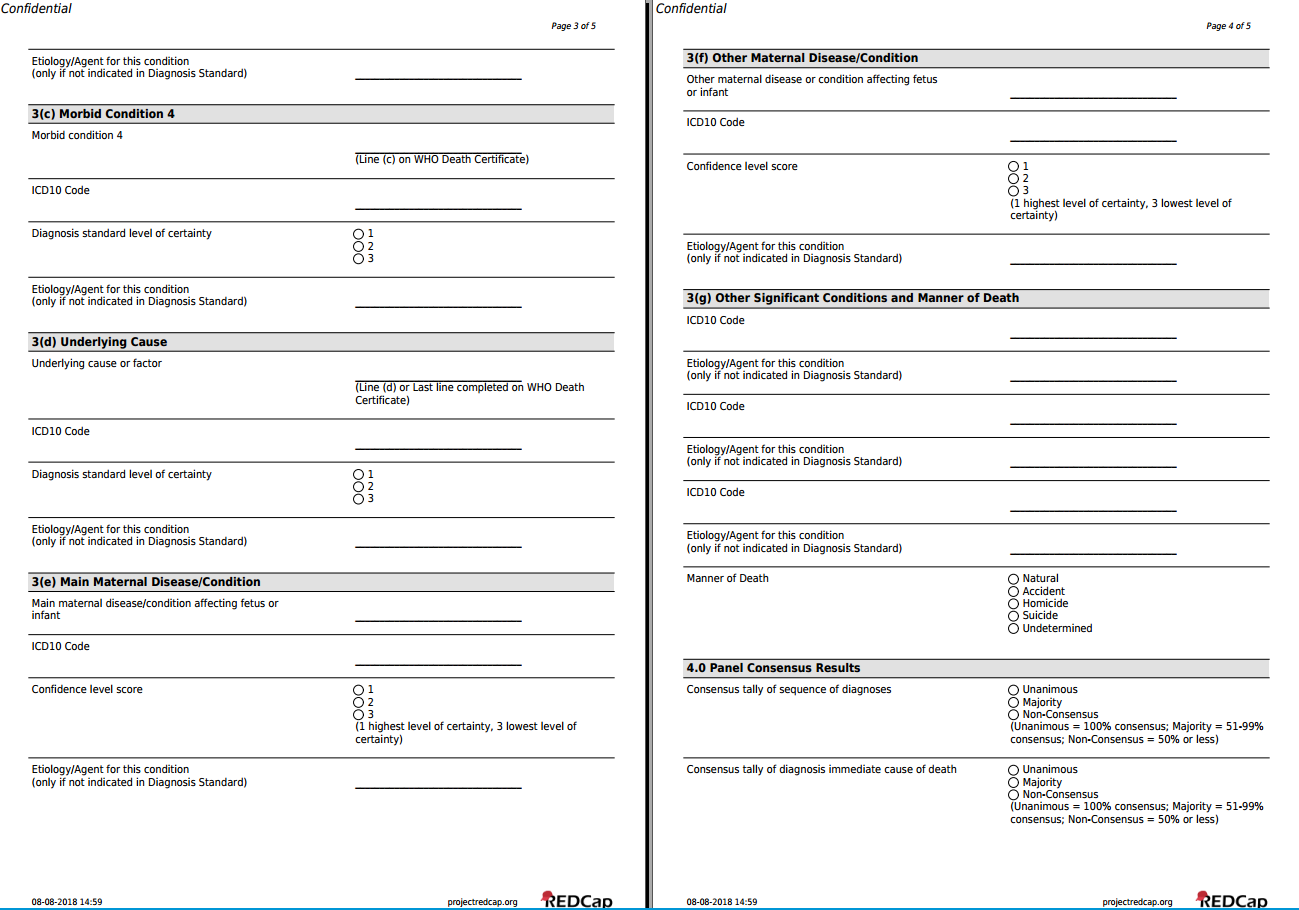


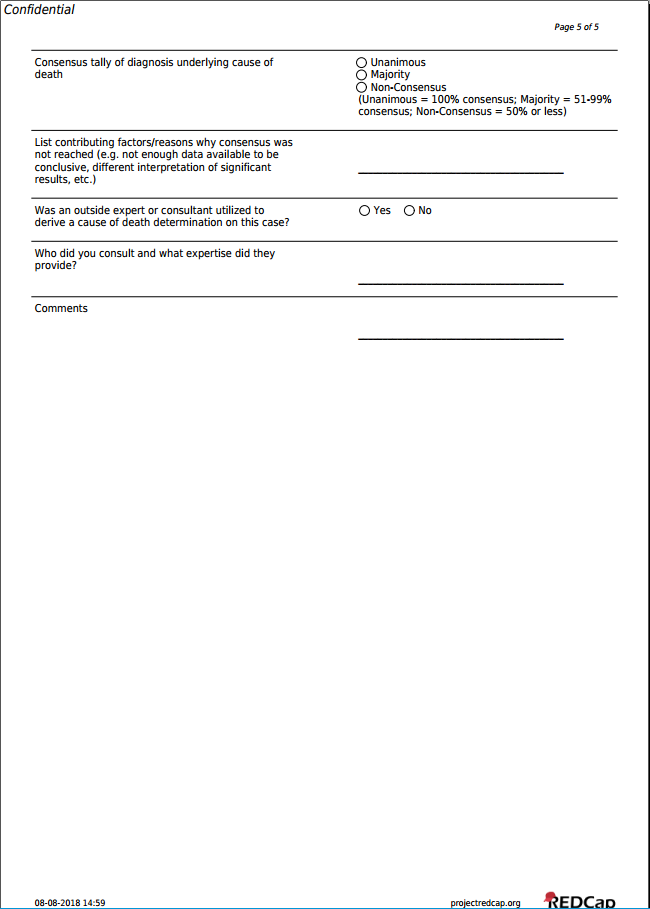


**Supplementary Table 3: Sample adequacy for histological evaluation**

| **Sample** | **Adequate** | **Autolysed** | **Inadequate** | **Suboptimal** | **Sum** |
| --- | --- | --- | --- | --- | --- |
| Liver | 137 (90.7) | 2 (1.3) | 8 (5.3) | 4 (2.6) | 151 (100) |
| Left lung | 96 (64) | 1 (0.7) | 35 (23.3) | 18 (12) | 150 (100) |
| Right lung | 104 (68.4) | 1 (0.7) | 28 (18.4) | 19 (12.5) | 152 (100) |
| Brain | 127 (84.1) | 0 (0) | 14 (9.3) | 10 (6.6) | 151 (100) |
| Total | **464 (76.8)** | **4 (0.6)** | **85 (14.1)** | **51 (8,4)** | 604 (100) |

**Supplementary Table 4a: Level of certainty for underlying cause of death attribution by the DeCoDe panel**

| **Underlying Cause of Death (ICD-PM)^a^** | **N** | **Level of Certainty** | | |
| --- | --- | --- | --- | --- |
|  |  | **Level 1**  **(Confident)** | **Level 2**  **(Probable)** | **Level 3**  **(Uncertain)** |
| Congenital malformations, deformations and chromosomal disorder (N1) | 20 | 17 (85.0%) | 2 (10.0%) | 1 (5.0%) |
| Complications of intrapartum events (N4) | 23 | 19 (82.6%) | 4 (17.4%) | 0 (0.0%) |
| Convulsions and disorders of cerebral status (N5) | 1 | 0 (0.0%) | 1 (100%) | 0 (0.0%) |
| Infection (N6) | 15 | 9 (60.0%) | 5 (33.3%) | 1 (6.7%) |
| Respiratory and cardiovascular disorders (N7) | 5 | 5 (100%) | 0 (0.0%) | 0 (0.0%) |
| Other neonatal conditions (N8) | 7 | 4 (57.1%) | 3 (42.9%) | 0 (0.0%) |
| Low birth weight/prematurity complications (N9) | 81 | 81 (100.0%) | 0 (0.0%) | 0 (0.0%) |
| Neonatal death of unspecified cause (N11) | 1 | 0 (0.0%) | 0 (0.0%) | 1 (100%) |
| Total | 153 | 135 (88.2%) | 15 (9.8%) | 3 (2.0%) |

**^a^** WHO ICD10 Perinatal Mortality (ICD-PM)

**Supplementary Table 4b: Level of certainty for immediate cause of death attribution by the DeCoDe panel.**

|  | Immediate Cause of Death | | N | Level of Certainty | | |
| --- | --- | --- | --- | --- | --- | --- |
|  |  | |  | Level 1 | Level 2 | Level 3 |
| Communicable (Infections) | Meningitis | Community-acquired | 3 | 1 (33.3%) | 1 (33.3%) | 1 (33.3%) |
|  |  | Nosocomial-acquired | 5 | 5 (100%) | 0 (0.0%) | 0 (0.0%) |
|  | Pneumonia | Community-acquired | 5 | 5 (100%) | 0 (0.0%) | 0 (0.0%) |
|  |  | Nosocomial-acquired | 28 | 27 (96.4%) | 1 (3.4%) | 0 (0.0%) |
|  | Sepsis | Community-acquired | 11 | 8 (72.7%) | 3 (27.3%) | 0 (0.0%) |
|  |  | Nosocomial-acquired | 32 | 30 (96.8%) | 1 (3.1%) | 1 (3.1%) |
|  | Pulmonary mucormycosis | | 1 | 1 (100%) | 0 (0.0%) | 0 (0.0%) |
| Non-communicable | Acute kidney failure | | 1 | 1 (100%) | 0 (0.0%) | 0 (0.0%) |
|  | Acquire hydrocephalus of the newborn | | 1 | 1 (100%) | 0 (0.0%) | 0 (0.0%) |
|  | Birth asphyxia | | 3 | 3 (100%) | 0 (0.0%) | 0 (0.0%) |
|  | Congenital malformations | | 7 | 7 (100%) | 0 (0.0%) | 0 (0.0%) |
|  | Hyaline membrane disease | | 14 | 13 (92.9%) | 1 (7.1%) | 0 (0.0%) |
|  | Hypoxic ischaemic encephalopathy | | 18 | 14 (77.8%) | 4 (22.2%) | 0 (0.0%) |
|  | Intrauterine hypoxia | | 6 | 5 (83.3%) | 1 (16.7%) | 0 (0.0%) |
|  | Intraventricular Haemorrhage | | 4 | 4 (100%) | 0 (0.0%) | 0 (0.0%) |
|  | Kernicterus | | 3 | 2 (66.7%) | 1 (33.3%) | 0 (0.0%) |
|  | Necrotising Enterocolitis | | 2 | 2 (100%) | 0 (0.0%) | 0 (0.0%) |
|  | Persistent foetal circulation | | 4 | 3 (75.0%) | 1 (25.0%) | 0 (0.0%) |
|  | Pneumothorax | | 1 | 1 (100%) | 0 (0.0%) | 0 (0.0%) |
|  | Pulmonary haemorrhage | | 1 | 1 (100%) | 0 (0.0%) | 0 (0.0%) |
|  | Shock | | 1 | 1 (100%) | 0 (0.0%) | 0 (0.0%) |
|  | Subarachnoid haemorrhage | | 1 | 1 (100%) | 0 (0.0%) | 0 (0.0%) |
|  | Vascular disorders of intestines | | 1 | 1 (100%) | 0 (0.0%) | 0 (0.0%) |
|  | Total | | 153 | 137 (89.5%) | 14 (9.2%) | 2 (1.3%) |

**Supplementary Table 5: Prevalence of antibiotic resistance of bacteria cultured post-mortem attributed to be either the immediate or underlying cause of death**

| **Antimicrobial** | ***Acinetobacter baumannii*** | ***Escherichia coli*** | ***Streptococcus agalactiae*** | ***Klebsiella pneumoniae*** | ***Staphylococcus aureus*** | *Enterobacter cloacae* | ***Enterococcus faecalis/faecium*** |
| --- | --- | --- | --- | --- | --- | --- | --- |
| Amikacin | 37/43 (86.0%) | 0/8 (0.0%) | ND | 1/25 (4.0%) | ND | 0/6 (0.0%) | ND |
| Amoxicillin.clavulanic.acid | ND | 2/8 (25.0%) | ND | 21/25 (84.0%) | ND | 7/8 (87.5%) | ND |
| Ampicillin.amoxicillin | ND | 8/8 (100%) | ND | 25/25 (100%) | ND | 8/8 (100%) | 7/14 (50.0%) |
| Cefazolin | ND | 1/8 (12.5%) | ND | 21/26 (80.8%) | ND | 7/8 (87.5%) | ND |
| Cefepime | 41/47 (87.2%) | 0/8 (0.0%) | ND | 19/26 (73.1%) | ND | 2/7 (28.6%) | ND |
| Cefotaxime, ceftriaxone | ND | 0/8 (0.0%) | 0/3 (0.0%) | 17/23 (73.9%) | ND | 3/8 (37.5%) | ND |
| Cefoxitin | ND | 0/8 (0.0%) | ND | 1/25 (4.0%) | ND | 7/8 (87.5%) | ND |
| Ceftazidime | 42/47 (89.4%)^1^ | 0/8 (0.0%) | ND | 20/26 (76.9%) | ND | 3/8 (37.5%) | ND |
| Cefuroxime, parenteral | ND | 0/8 (0.0%) | ND | 21/26 (80.8%) | ND | 4/8 (50.0%) | ND |
| Chloramphenicol | ND | 0/8 (0.0%) | 0/3 (0.0%) | 8/26 (30.8%) | 13/15 (86.7%) | 2/7 (28.6%) | ND |
| Ciprofloxacin | 46/47 (97.9%)^2^ | 4/8 (50.0%) | ND | 4/24 (16.7%)^1^ | 11/14 (78.6%) | 0/8 (0.0%) | ND |
| Clindamycin | ND | ND | 0/3 (0.0%) | ND | 2/15 (13.3%) | ND | ND |
| Cloxacillin | ND | ND | ND | ND | 13/15 (86.7%) | ND | ND |
| Colistin | 0/47 (0%) | ND | ND | ND | ND | ND | ND |
| Ertapenem | ND | 0/8 (0.0%) | ND | 0/26 (0.0%) | ND | 0/8 (0.0%) | ND |
| Erythromycin.azithromycin | ND | ND | 0/3 (0.0%) | ND | 13/15 (86.7%) | ND | ND |
| Fusidic_acid | ND | ND | ND | ND | 0/15 (0.0%) | ND | ND |
| Gentamicin | 47/47 (100%) | 4/8 (50.0%) | ND | 22/26 (84.6%) | 13/15 (86.7%) | 3/7 (42.9%) | ND |
| Gentamicin, high.level.resistance | ND | ND | ND | ND | ND | ND | 7/15 (46.7%) |
| Imipenem | 46/46 (100%) | 0/8 (0.0%) | ND | 0/26 (0.0%) | ND | 0/8 (0.0%) | ND |
| Linezolid | ND | ND | 0/3 (0.0%) | ND | 1/15 (6.7%) | ND | 0/15 (0.0%) |
| Meropenem | 46/46 (100%) | 0/8 (0.0%) | ND | 0/25 (0.0%) | ND | 0/8 (0.0%) | ND |
| Nalidixic acid | ND | 5/8 (62.5%) | ND | 3/26 (11.5%) | ND | 1/8 (12.5%) | ND |
| Penicillin.ampicillin | ND | ND | 0/3 (0.0%) | ND | 15/15 (100%) | NDS | ND |
| Piperacillin.tazobactam | 45/45 (100%) | 0/8 (0.0%) | ND | 8/26 (30.8%)^3^ | ND | 0/8 (0.0%) | ND |
| Rifampicin | ND | ND | ND | ND | 0/15 (0.0%) | ND | ND |
| Streptomycin, high.level.resistance | ND | ND | ND | ND | ND | ND | 2/15 (13.3%) |
| Tobramycin | 39/46 (84.8%)^1^ | 3/8 (37.5%) | ND | 18/26 (69.2%) | ND | 2/8 (25.0%) | ND |
| Trimethoprim.sulfamethoxazole | 46/46 (100%) | 6/8 (75.0%) | ND | 22/26 (84.6%) | 13/15 (86.7%) | 3/8 (37.5%) | ND |
| Vancomycin | ND | ND | 0/3 (0.0%) | ND | 0/15 (0.0%) | ND | 0/16 (0.0%) |

^1^One intermediate resistance. ^2^Two intermediate resistance. ^3^Four intermediate resistance.

ND= not done

**References**

1. Moyo F, Haeri Mazanderani A, Barron P, et al. Introduction of Routine HIV Birth Testing in the South African National Consolidated Guidelines. Pediatr Infect Dis J **2018**;

2. Madhi SA, Cutland CL, Kuwanda L, et al. Influenza Vaccination of Pregnant Women and Protection of Their Infants. N Engl J Med **2014**; 371:2340. Available at: https://doi.org/10.1056/NEJMc1412050.

3. Liu L, Oza S, Hogan D, et al. Global, regional, and national causes of under-5 mortality in 2000–15: an updated systematic analysis with implications for the Sustainable Development Goals. Lancet **2016**;

4. World Health Organization. The WHO application of ICD-10 to deaths during the perinatal period. 2016: 1–88. Available at: http://www.who.int/reproductivehealth/publications/monitoring/icd-10-perinatal-deaths/en/. Accessed 29 August 2018.
