# Supplementary figures and images for: Unraveling Specific Causes of Neonatal Mortality Using Minimally Invasive Tissue Sampling: An Observational Study
Source: Clin Infect Dis. 2019 Oct 9;69(Suppl 4):S351–60. doi: 10.1093/cid/ciz574 (PMC6785687; doi:10.1093/cid/ciz574)

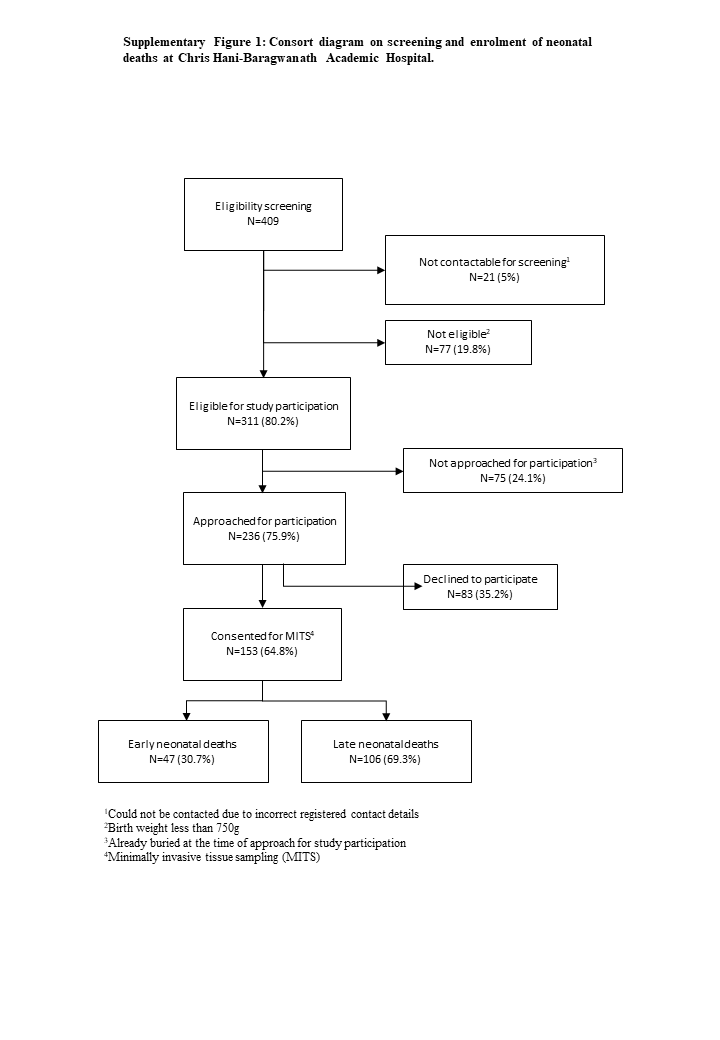

Supplement: ciz574_suppl_Supplementary_Figure-1 [file ciz574_suppl_supplementary_figure-1.png]
